# Microscopy-based techniques for studying the material properties of biomolecular condensates in the cellular environment

**DOI:** 10.1007/s12551-025-01343-5

**Published:** 2025-07-28

**Authors:** Tin Long Chris Ng, Luisa Capalbo, Janet R. Kumita

**Affiliations:** Department of Pharmacology, University of Cambridge, Tennis Court Road, Cambridge, CB2 1PD UK

**Keywords:** Phase separation, Biomolecular condensates, Material properties, Microscopy-based characterisation

## Abstract

The material properties of biomolecular condensates, such as interfacial tension, viscoelasticity, stiffness, and molecular dynamics, are crucial for their biological functions in processes like signal transduction, stress response, and gene regulation. These properties influence both endogenous condensates, like the nucleolus and stress granules, and synthetic condensates engineered for potential drug delivery applications. In vitro studies, using purified components, provide controlled environments to explore the fundamental physics of phase separation, offering high precision in manipulating molecular components and conditions. However, cell-based characterisations are indispensable for understanding the physiological relevance of biomolecular condensates, accounting for molecular crowding, post-translational modifications, and interactions with cellular structures. Light-microscopy techniques offer the potential to bridge in vitro findings with *in cellulo* behaviour. This review outlines some fundamental challenges of *in cellulo* studies and discusses the potential of fluorescently labelling biomolecular condensates using the tetracysteine tag/biarsenical dye strategy. We describe how fluorescence-based techniques, including fluorescence recovery after photobleaching (FRAP) and emerging techniques like fluorescence lifetime imaging microscopy (FLIM), flicker spectroscopy, and raster image correlation spectroscopy (RICS), may be used to gain a detailed understanding of the material properties of biomolecular condensates within the cellular environment. Finally, we discuss the potential of Brillouin light scattering (BLS) microscopy, a label-free technique that holds potential for deciphering the cellular biophysics of biomolecular condensates.

## Introduction

**Fig. 1:**
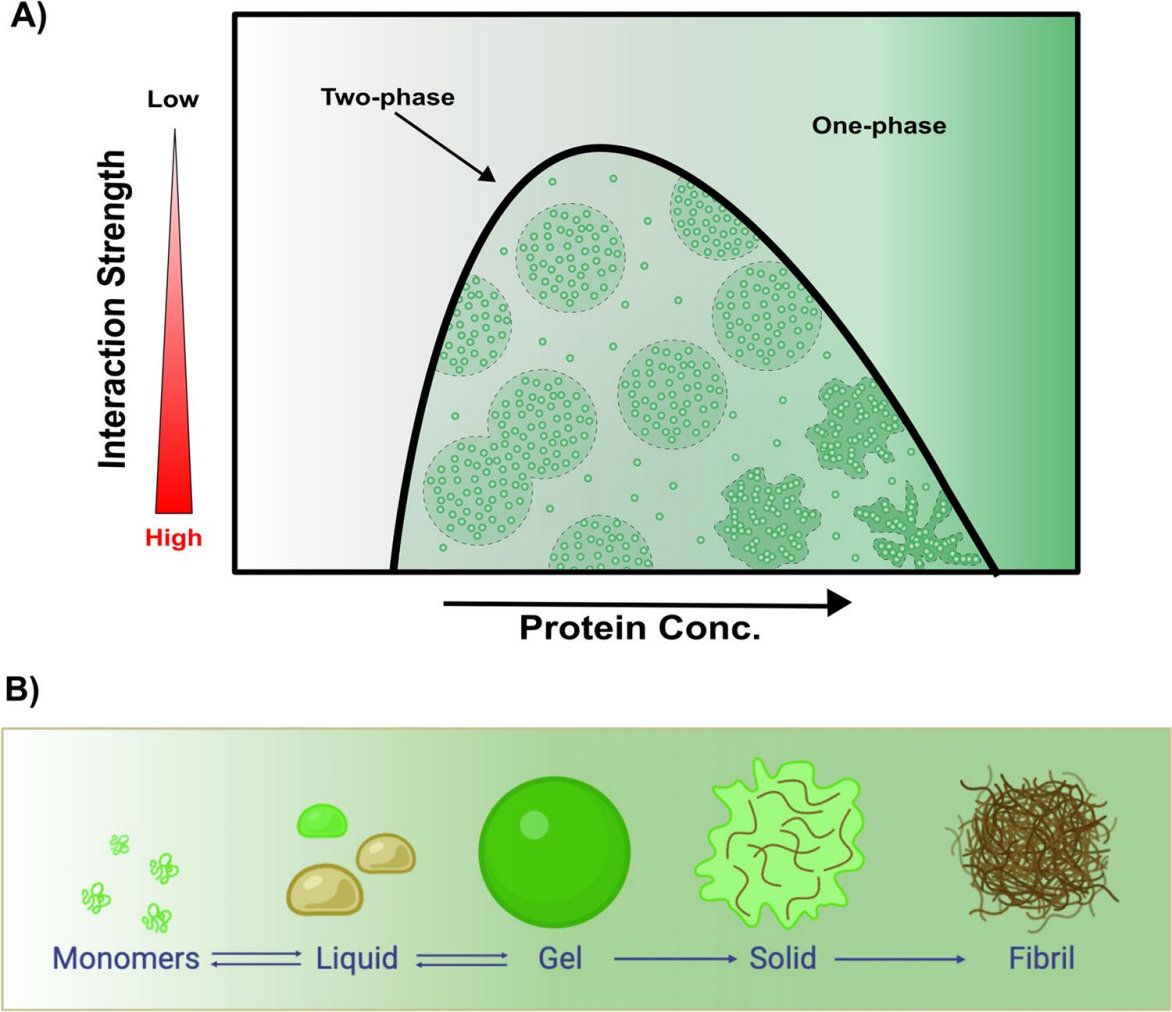
Phase diagram and material state transitions of biomolecular condensates. (**A**) Schematic phase diagram illustrating how intermolecular interactions and protein concentration influence phase separation. Increasing interaction strength and protein concentration drive the system from a homogeneous one-phase state to a two-phase regime, where biomolecular condensates form. (**B**) Phase separation enables monomers to assemble into liquid-like condensates, which may further mature into gel-like or glass-like structures before eventually forming solid fibrils. While direct conversion from a liquid state to fibrillar aggregates is possible, it is not depicted here for simplicity. (**B**) was created using https://BioRender.com

The organisation of biomolecular condensates through membraneless compartmentalisation has emerged as a fundamental regulatory mechanism within cells, enabling the spatial and temporal control of biochemical reactions (Banani et al. 2017; Brangwynne et al. 2009). These condensates, formed through phase separation, provide dynamic microenvironments that concentrate different biomolecules to facilitate cellular functions such as signal transduction, RNA metabolism, and stress responses (Lyon et al. 2021; Shin and Brangwynne 2017). Phase separation is a thermodynamically favourable process in which the strength of molecular interactions between molecules is stronger than interactions between the molecules and the solvent. This results in a transition of macromolecules from a single phase to a two-state liquid droplet phase (Fig. 1A) (Woodruff et al. 2018). Over time, these condensates may evolve, shifting from a dynamic liquid-like state to a more rigid, solid-like structure (Fig. 1B). Several intrinsically disordered regions (IDR)-containing proteins, such as FUS, hnRNPA1, Whi3, and fibrillarin, have been shown to undergo phase separation initially as liquid droplets, which can subsequently transition toward more stable, solid-like assemblies (Courchaine et al. 2016; Mitrea et al. 2022). The physical properties of condensates depend on the molecular interactions and environmental conditions that regulate these critical transitions. In nature, macromolecules that undergo phase separation are usually enriched in multivalent, modular domains or IDRs. They contain an array of proteins and nucleic acids, and typically, these biomolecules can be divided into two categories — “scaffolds” and “clients” (Banani et al. 2016). Scaffolds are components that are essential for structural integrity and drive phase separation through multivalent interactions, whereas clients are dispensable for the assembly of condensates but are recruited to the condensates under certain conditions through interactions with the scaffolds (Choi et al. 2020; Martin et al. 2020). It is becoming increasingly clear that, although scaffold regions drive condensate formation, the ultimate material state of the condensate is tuned by the client interactions (Harmon et al. 2017; Woodruff et al. 2018). Importantly, the material properties of biomolecular condensates—such as interfacial tension, viscoelasticity and molecular dynamics—govern their formation, stability, and ability to regulate biochemical processes and molecular interactions within cells (Jawerth et al. 2018; Sun et al. 2024).

Endogenous biomolecular condensates have been extensively characterised, including stress granules, P bodies, nucleoli, and transcriptional assemblies (Brangwynne et al. 2009; Kedersha et al. 2005). The functional versatility of biomolecular condensates and the emergent properties of compartmentalisation in the dense phase have also garnered significant interest in synthetic biology (Faltova et al. 2018; Guan et al. 2021; Lasker et al. 2022; Simon et al. 2019). Consequently, engineered condensate systems have been developed to reconstitute biomolecular condensates in simplified settings, allowing for controlled investigation of phase separation principles and the application of condensate-based technologies in biotechnology and therapeutic design (Dai et al. 2023; Shin and Brangwynne 2017). Moreover, these synthetic condensate systems provide a way to bridge the in vitro physicochemical properties of these systems, composed of purified components, with behaviour in cell models. This is particularly advantageous as techniques for quantifying the material properties of condensates within cells are limited despite the importance of studying them in the complex cellular environments, where interactions with other organelles, molecular crowding, and post-translational modifications may play a crucial role (Patel et al. 2015).

Despite significant research efforts, the role of material properties in regulating condensate function remains unclear (Sundaravadivelu Devarajan et al. 2024). Studies suggest that evolutionary selection may have influenced specific material properties of condensates, requiring a deeper understanding of how primary sequence features determine physical characteristics and functional roles (Alberti et al. 2019). While in vitro measurements of condensate material properties have achieved high precision (Alshareedah et al. 2021a, b; Linsenmeier et al. 2022), emerging evidence suggests that these properties are not solely dictated by condensate composition but are also influenced by external factors such as ionic strength and medium stress. For instance, while salt concentration significantly influences surface tension, it does not affect the intrinsic phase separation propensity of the condensates, highlighting the specific role of the surrounding medium in modulating interfacial properties (Jawerth et al. 2018).

To systematically understand how material properties influence biomolecular condensates, it is necessary to integrate approaches from multiple experimental and computational paradigms. Recent studies have employed a combination of in vitro reconstitution, *in cellulo* experiments, and in silico simulations to investigate the biophysical principles underlying condensate formation and behaviour (Garabedian et al. 2022, 2021; Lasker et al. 2022; Ray et al. 2020). Fluorescence microscopy remains the predominant method for assessing condensate dynamics, with recent advancements incorporating optical tweezers and microrheology techniques to probe mechanical properties in vitro (Alshareedah et al. 2021a, b; Shen et al. 2023; Wei et al. 2017). Nevertheless, due to both technological and practical limitations, obtaining high-quality qualitative and quantitative data remains challenging. This has resulted in inconsistencies across studies, particularly in cellular systems, where the complexity of the intracellular environment poses additional difficulties in accurately assessing material properties (Hoang et al. 2024). Consequently, a comprehensive framework aligning in vitro and in cell measurements is still lacking. Given these challenges, there is an increasing need for alternative and advanced techniques that mitigate the drawbacks of fluorescence-based methods by allowing direct, non-perturbative measurements of condensate properties.

In this review, we aim to define key criteria for the accurate measurement of condensate material properties in cellular systems and discuss the use of the tetracysteine (TC) tag/biarsenical dye system as a versatile, minimally perturbed fluorescent-labelling strategy for biomolecular condensates. Next, we discuss how light microscopy techniques can overcome certain challenges for measuring material properties in cells and provide an overview of microscopy-based techniques used to study condensate mechanics, highlighting their respective advantages and limitations both in vitro and in the cellular context. In particular, we discuss emerging techniques such as fluorescence lifetime imaging microscopy (FLIM), flicker spectroscopy, and Brillouin light scattering (BLS) microscopy, and how they may potentially bridge the gap in relating in vitro and *in cellulo* biophysical measurements.

### Selection and handling of fluorescent tags

Fluorescent labelling is a crucial tool for studying biomolecular condensates, enabling real-time tracking and analysis in both in vitro and live cell environments. While fluorescent proteins (FPs) are commonly used due to their stability and high signal intensity (Cranfill et al. 2016), recent research suggests that their incorporation can significantly influence condensate properties, affecting phase separation behaviour, aggregation tendencies, and molecular interactions (Dörner et al. 2024). The pH sensitivity, charge, and oligomerisation state of FPs and chemical dyes can also lead to artefactual recruitment or exclusion of biomolecules within condensates, resulting in misleading diffusion and viscosity measurements (Barkley et al. 2024; Dörner et al. 2024). Studies have also shown that tags like EGFP and mCherry exhibit differential effects on condensate size and dynamics. Besides, excluded volume effects from large FP tags may alter material properties like viscosity and diffusion (Dörner et al. 2024; Mitrea et al. 2018a, b). Moreover, the accumulation of fluorescent dyes within the dense phase may distort the apparent concentration gradient between dense and dilute phases, complicating accurate quantification.

**Fig. 2:**
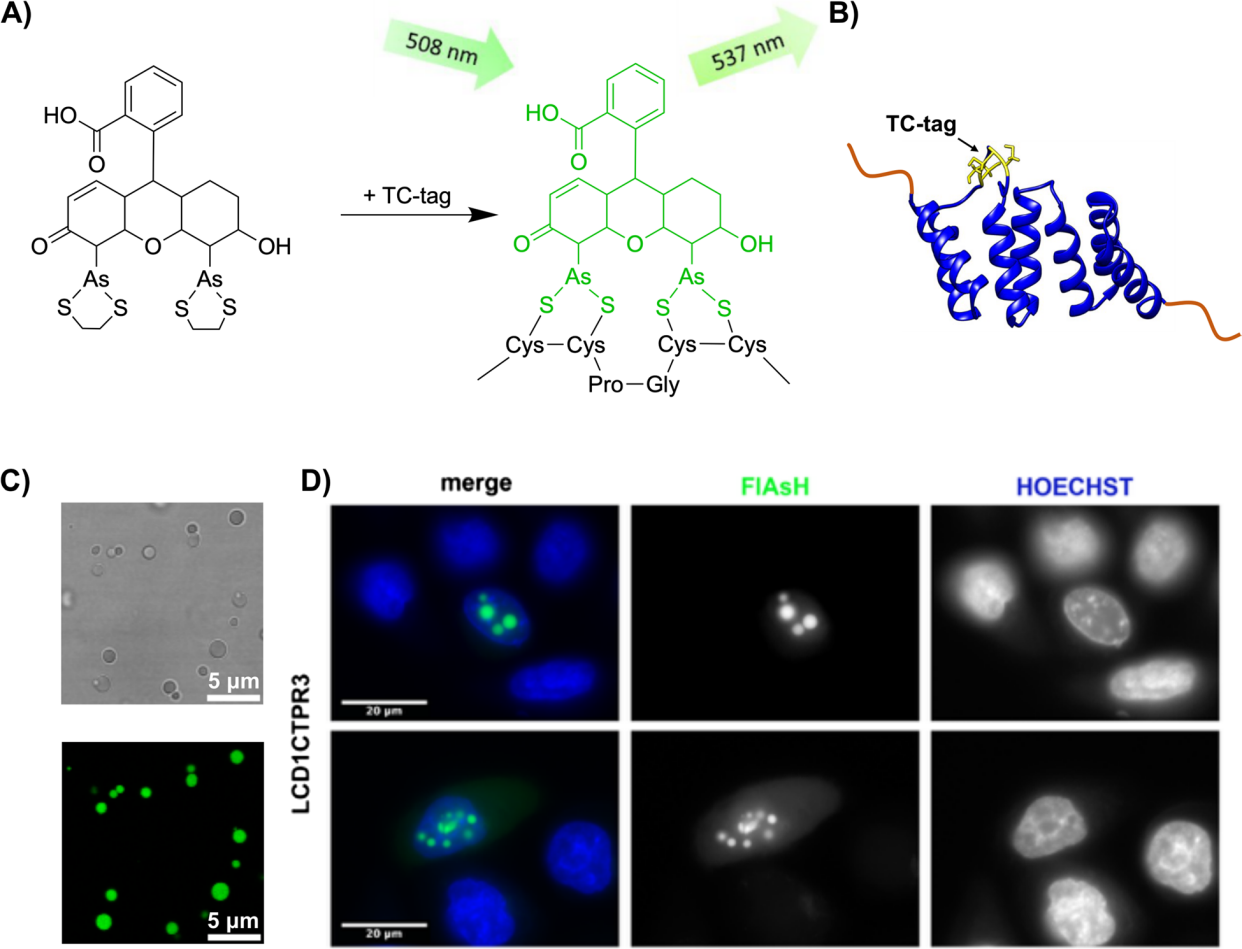
Fluorescence labelling of TC-tagged consensus tetratricopeptide repeat (CTPR) condensates using FlAsH-EDT_2_. (**A**) Schematic representation of FlAsH-EDT_2_ binding to a tetracysteine (TC) tag, resulting in fluorescence activation. (**B**) Illustration of a TC-tag motif (yellow) grafted onto a CTPR protein (blue) that has been engineered with phase-separating low complexity domains (orange) (Ng et al. 2025). (**C**) In vitro biomolecular condensates of TC-tagged CTPR proteins. (Top) Brightfield image showing the presence of phase-separated condensates LCD1-CTPR3. (Bottom) Confocal fluorescence microscopy image of FlAsH-labelled condensates visualised at 488 nm. Scale bar: 5 µm (Ng et al. 2025). (**D**) Live-cell imaging of TC-tagged CTPR condensates. HeLa cells were transiently transfected with the TC-tagged LCD1-CTPR3 constructs. Biomolecular condensates within the cells were visualised using FlAsH fluorescence (middle panel) and Hoechst for nuclear staining (right panel). Scale bar: 20 µm (unpublished data)

Although there is an increase towards using small, fluorescent dyes to selectively label condensates in vitro, traditional strategies targeting specific reactions with amino acid side chains (i.e. through amine or thiol-related chemistry) cannot be used to specifically label the condensates inside the cell. A novel covalent-labelling strategy for visualising proteins inside cells was developed by Tsien and co-workers (Griffin et al. 1998). This fluorescent, membrane-permeable biarsenical compound derived from fluorescein, called ﻿Fluorescein Arsenical Hairpin Binder (FlAsH-EDT_2_) was designed to bind covalently to tetracysteine motifs ﻿Cys-Cys-Xaa-Xaa-Cys-Cys (CCXXCC, where X denotes any amino acid) engineered into target proteins (Fig. 2A) (Griffin et al. 1998). It becomes fluorescent upon specific binding, making it ideal for visualising proteins in living cells (Ng et al. 2019). Its small size and ability to specifically label target proteins make FlAsH-EDT_2_ an alternative option for studying dynamic cellular structures like biomolecular condensates in vitro and *in cellulo* (Ng et al. 2025; Ray et al. 2020)*.*

The tetracysteine motif can be grafted into target proteins within different structural elements, often not only incorporated as a linear sequence at a protein’s terminal end (Adams et al. 2002; Ray et al. 2020), but also embedded into α-helical conformations (Griffin et al. 1998; Tsytlonok and Itzhaki 2012) or within loop regions of a protein, such as within our consensus tetratricopeptide repeat (CTPR) condensate system (Fig. 2B–D) (Ng et al. 2025). This flexibility in placement is particularly advantageous for condensate research, where the tag’s positioning could influence its accessibility to FlAsH-EDT_2_ in crowded environments (Fatti et al. 2025). An additional advantage of the tetracysteine tag is that it can be used to monitor intermolecular interactions by engineering half of the sequence into one protein and the other half on the potential binding partner (Krishnan and Gierasch 2008; Luedtke et al. 2007). If the proteins are in close contact to reconstitute the full TC-tag geometry, then fluorescent labelling will occur. This has been termed as bipartite or split tetracysteine motifs and has been successfully used to detect intermolecular coiled-coil formation in live cells (Luedtke et al. 2007) and as an intramolecular probe to evaluate conformational changes within the beta-strand core of a protein (Krishnan and Gierasch 2008).

A key consideration when using TC-tag labelling is the redox state of the surrounding environment. For in vitro assays, the presence of free cysteine residues within the TC motif may lead to undesired disulphide bond formation under oxidative conditions, with such intermolecular crosslinking potentially altering condensate properties. Therefore, it is important to carefully optimise the concentration of reducing agents (e.g. dithiothreitol (DTT) or tris(2-carboxyethyl) phosphine (TCEP)) to maintain a fully reduced state throughout the assay. Recent work using designed synthetic peptides that can form intermolecular cross-links within biomolecular condensates has shown that subtle changes in redox conditions during in vitro assays can influence the material properties and phase behaviour of condensates, including changes in viscoelastic properties (Mondal et al. 2024). It is noted that such issues are less likely to occur in the cellular context, given the reducing nature of the cytosolic environment.

To minimise perturbation of condensate biology, the size of the fluorescent tag is a critical parameter when selecting a labelling strategy. Besides the TC-tag, other peptide-based systems offer attractive alternatives. For example, click chemistry enables rapid, site-specific fluorescent tagging of proteins through the incorporation of unnatural amino acids, yielding minimal steric interference due to the very small size of the functional group. This technique has gained traction owing to its high labelling efficiency, bioorthogonality, and compatibility with both in vitro and live cell environments. It supports modular, stoichiometric labelling and has been applied successfully to high-resolution microscopy (Raulf et al. 2014). The same chemistry is now beginning to migrate into condensate research, where its negligible steric footprint and low background have clear advantages (Fu et al. 2024). Another emerging candidate is the ALFA-tag, a 13–amino acid helical epitope recognised by a high-affinity nanobody. The ALFA-tag system enables minimal background labelling and has been effectively deployed for live-cell imaging, immunoprecipitation, and super-resolution microscopy (Götzke et al. 2019). Mumford and co-workers recently used an ALFA × 4 cassette to reveal nanometre-scale client clusters that conventional microscopy was unable to resolve (Mumford et al. 2024). The need to add the ≈15 kDa nanobody, however, introduces extra mass that could, in principle, alter condensate material properties. Recent research found that even conventional N-terminal epitope tags can measurably shift the phase behaviour of condensate-forming proteins (Dao et al. 2024). Taken together, click-handles and the ALFA system offer promising, low-perturbation routes for dissecting condensate composition and nanoscale organisation, but their application to condensate biology is still at an early stage and will require careful, case-by-case validation.

Despite the smaller size, chemical dyes can still potentially alter condensate dynamics (Fatti et al. 2025); therefore, when using fluorescent dyes, the minimal effective concentration should be applied to reduce perturbations and maintain condensate properties as close to their native state as possible. It is suggested that a ratio of 1–2% of labelled-to-unlabelled species is considered good practice to reduce artefactual effects (Alshareedah et al. 2021a, b). Another critical consideration in fluorescence-based imaging is phototoxicity, which arises from prolonged exposure to high-intensity light. Excessive illumination can disrupt condensate integrity, induce oxidative stress, and lead to phase instability (Banani et al. 2017). Damage to cellular macromolecules due to excitation light illumination can impair cell physiology and even lead to cell death (Icha et al. 2017). These limitations highlight the importance of selecting imaging modalities that minimise phototoxicity while maintaining high accuracy in molecular diffusion measurements. Integrating lower intensity illumination strategies or emerging label-free imaging techniques may help mitigate these artefacts while preserving condensate dynamics.

### Criteria for material property studies in cells

Accurate measurement of the material properties of condensates in live cell environments is crucial for understanding their phase behaviour, dynamics, and function (Hoang et al. 2024). Experimental approaches must account for these factors to obtain physiologically relevant measurements. Unlike in vitro reconstitution systems, cellular condensates are compositionally heterogeneous, influenced by molecular crowding and the distinct physicochemical properties of their dense phase microenvironments (Hatters 2023; Mitrea et al. 2018a, b)*.* Stress granules and P-bodies, for example, contain phase-separated subdomains with varying viscosity and molecular exchange rates (Banjade and Rosen 2014)*.* Moreover, the condensate’s internal molecular diffusion is affected by short-lived electrostatic interactions, enabling rapid internal rearrangements despite high overall viscosity (Galvanetto et al. 2023). Imaging techniques must therefore resolve molecular exchange rates and subdomain differences to ensure *in cellulo* characterisation accuracy (Gao et al. 2025).

Condensates are highly dynamic, undergoing rapid changes in size, shape, and composition (Galvanetto et al. 2023). Studying these transient processes requires techniques with sufficient temporal resolution, particularly for capturing fusion, dissolution, and assembly events occurring over milliseconds to minutes (Galvanetto et al. 2023). Inadequate sampling may misrepresent condensates as more gel-like than they truly are. Additionally, condensate properties vary across subcellular compartments, necessitating high spatial resolution to distinguish local biochemical influences from global cytoplasmic effects (Snead and Gladfelter 2019). For example, stress granule viscosity and diffusion rates change depending on cytoplasmic crowding conditions, making precise spatial tracking essential (Kitamura et al. 2023). By integrating high-speed, multi-modal imaging approaches, researchers can track condensate dynamics with minimal perturbation while maintaining high temporal and spatial fidelity in live cell environments.

### Techniques for studying material properties

A variety of biophysical techniques offer unique insights into condensate mechanics and have been employed to obtain reliable measurements of the material properties of condensates. By applying these techniques to both in vitro and *in cellulo* systems, researchers can better align the results obtained from different environments, providing a more comprehensive understanding of condensate behaviour in physiological settings. Below, we review key techniques that can be applied to both types of systems and used to probe condensate properties, comparing their strengths, weaknesses, and recent findings. We also discuss how combining these complementary techniques may provide a more complete understanding of condensate biophysics.

### Coalescence-based techniques

**Fig. 3:**
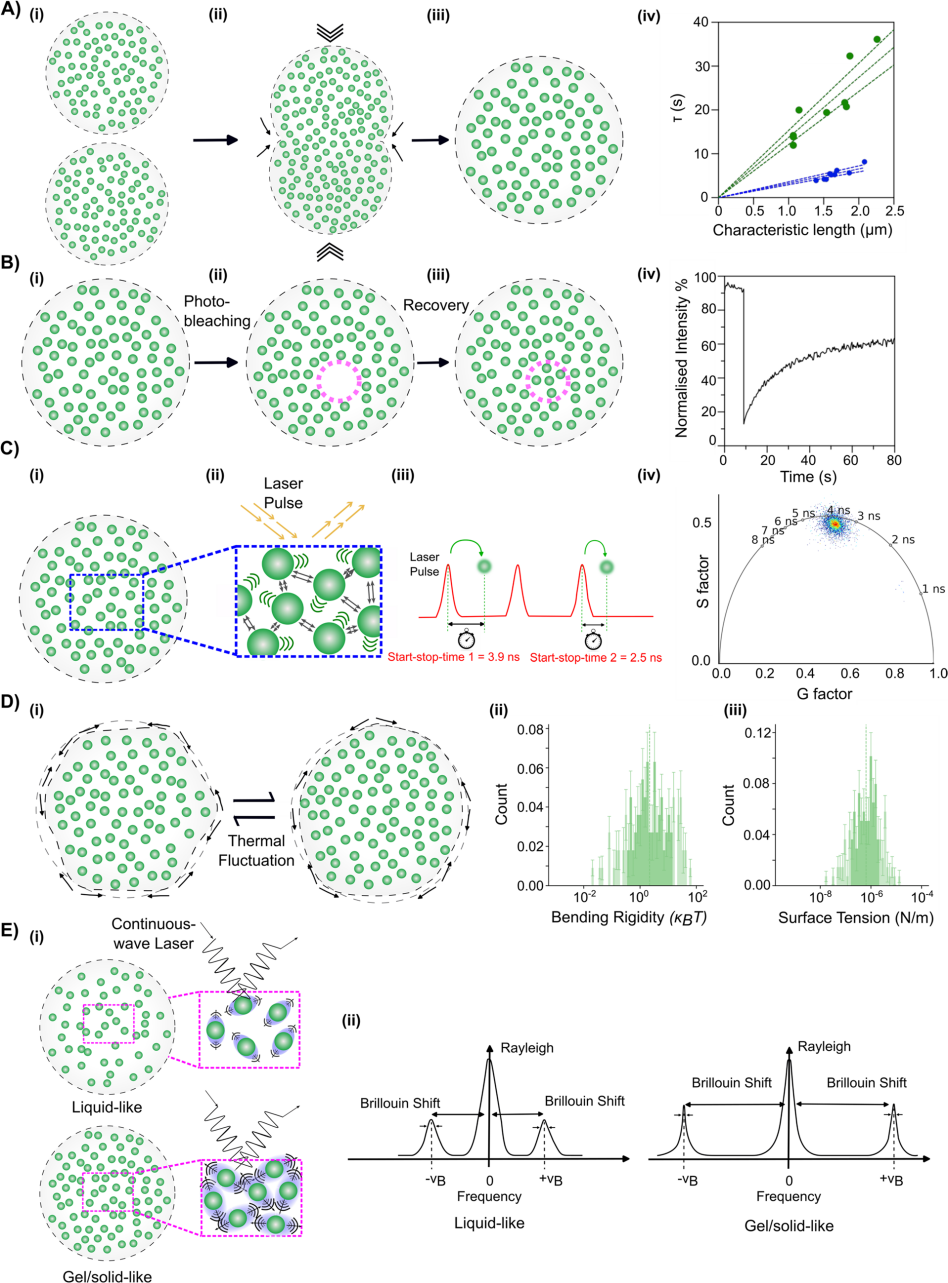
Schematics for techniques capable of measuring material properties of biomolecular condensates. (**A**) Coalescence-based assay: (i–iii) Coalescence dynamics of condensates are illustrated, demonstrating their ability to merge and equilibrate. More liquid-like condensates coalesce and relax into a spherical shape faster than a gel-like condensate. (iv) A representative graph of characteristic relaxation time vs. characteristic length (diameter) is shown. The inverse capillary velocity (ICV)**,** derived from the slope of the fitted line, provides insight into the material properties of condensates by assessing their fusion kinetics. Sample A (green) has an ICV of 13.71 s/μm and sample B (blue) has an ICV of 3.28 s/μm under identical buffer conditions. (**B**) Fluorescence recovery after photobleaching (FRAP): (i–ii) A region of interest (ROI) within a condensate (pink dashed circle) is photobleached, and (iii) subsequent fluorescence recovery is monitored to assess molecular mobility and exchange rates. (iv) A representative FRAP recovery curve, where the mobile fraction and recovery half-life are derived from fluorescence intensity changes over time. (**C**) Fluorescence lifetime imaging microscopy (FLIM) via time-correlated single-photon counting (TCSPC): (i) Molecular packing and interactions within condensates influence fluorescence lifetime. (ii) A laser pulse excites the sample, and the fluorescence lifetime of emitted photons is recorded at each pixel using TCSPC. (iii) Data is represented in a phasor plot, where each pixel’s fluorescence lifetime is decomposed into G and S factors, allowing visualisation of distinct molecular environments within the condensate. **(D**) Flicker spectroscopy for interfacial tension and bending rigidity: (i) Condensates undergo thermal fluctuations that deform their surface over time. The extent of these fluctuations depends on interfacial tension and bending rigidity. The flicker spectrum is extracted from a rapid sequence of images and mathematically transformed to infer the (ii) bending rigidity and (iii) interfacial tension. (**E**) Brillouin light scattering (BLS) microscopy: (i) The viscoelastic properties of condensates influence the interaction with phonons, generating distinct Brillouin frequency shifts upon excitation with a continuous-wave monochromatic laser. More liquid-like condensates generate larger shifts with broader linewidths due to increased compressibility, whereas gel/solid-like condensates exhibit shorter shifts and narrower linewidths due to higher elasticity and molecular rigidity. (ii) A representative Brillouin spectrum shows the frequency shift and linewidth variations between liquid-like and solid-like condensates, enabling the assessment of their mechanical properties in a non-contact, label-free manner. Data in A(iv), B(iv), C(iii), and D(ii and iii) are unpublished data

Coalescence-based techniques measure how condensates relax into spherical shapes after merging, a process driven by interfacial tension and resisted by viscosity. When two condensates coalesce, they initially adopt an ellipsoidal shape due to forming a capillary bridge at the point of contact. This structure then gradually relaxes back to a sphere as interfacial tension overcomes viscous resistance (Brangwynne et al 2011; Leal-Calderon et al. 2007). The time scale of this shape relaxation is governed by the balance of interfacial tension (which drives the droplet to minimise surface area) and viscosity (which resists flow). By analysing fusion events — either spontaneous or induced — one can estimate the ratio of interfacial tension (*γ*) to viscosity (*η*) from the relaxation time as a function of droplet size. This provides a readout of condensate material liquidity. This ratio, also known as the inverse capillary velocity (ICV), serves as a key parameter in describing the physical properties of the condensates (Fig. 3A) (Brangwynne et al. 2011; Elbaum-Garfinkle et al. 2015; Fabrini et al. 2024; Sato and Takinoue 2023).

Multiple advanced techniques based on this concept of observing natural ‘passive’ fusion events as well as using induced, ‘active’ coalescence or dissociation using optical tweezers, have been developed and utilised to estimate the ICV and the fusion force dynamics of biomolecular condensate systems (Alshareedahet al. 2021a, b; Ghosh & Zhou 2020; Jawerth et al. 2018; Patel et al. 2015). The optical tweezers set-up allows the trapping of two condensates, enabling controlled fusion under defined mechanical conditions. Active approaches offer greater experimental control and can manipulate condensate pairs with high spatial precision, allowing systematic exploration of size-dependent fusion dynamics. While traditionally restricted to in vitro systems, recent developments have extended the application of optical tweezers into live-cell environments such as zebrafish progenitor cells and in *Caenorhabditis elegans* tissue cells (Català-Castro et al. 2025). Nevertheless, such *in cellulo* implementations remain technically challenging, limited in scope, and present additional challenges due to intracellular molecular crowding and active cellular processes that influence fusion behaviour. As a result, the material parameters derived from these measurements often provide indirect insight into condensate behaviour under physiological conditions. Monitoring passive fusion events has been used to determine ICVs both in vitro and in cells (Brangwynne et al. 2009, 2011; Fabrini et al. 2024).

Although monitoring fusion events has advantages, such as being able to do this with label-free samples, these assays still present several fundamental limitations that must be acknowledged to ensure accurate data interpretation. Primarily, coalescence-based techniques often operate under the assumption that condensates behave as simple Newtonian fluids, characterised by a constant viscosity irrespective of the applied shear stress (Elbaum-Garfinkle et al. 2015). However, many biomolecular condensates exhibit viscoelastic properties, displaying both viscous and elastic behaviours (Jawerth et al. 2018). In such cases, the relaxation process is influenced by an increase in elastic energy due to compression, potentially leading to arrested coalescence, where droplets fail to fully merge and relax into a spherical shape (Ranganathan et al. 2022). This phenomenon has been observed in systems where elastic forces counteract the driving force of interfacial tension, hindering complete fusion (Ghosh et al. 2021; Linsenmeier et al. 2022). Besides, these assays yield only the ratio of interfacial tension to viscosity (*η*/*γ*), without providing absolute values for either parameter.

Despite the above challenges, coalescence-based measurements have yielded important insights. Low interfacial tensions have been confirmed across many condensate types (∼10^−7^ to 10^−4^ N/m), explaining why condensates round up and deform easily (Wang et al. 2021). For example, Zidovska and co-workers applied high-resolution fusion analysis to nucleoli in human cells, showing that nucleolar droplets coalesce with dynamics consistent with very low surface tension (Caragine et al. 2018).

### Fluorescence recovery after photobleaching (FRAP)

Fluorescence recovery after photobleaching (FRAP) is widely used to assess the fluidity and molecular exchange rates within condensates in both in vitro and *in cellulo* systems (Elbaum-Garfinkle et al. 2015; Li et al. 2012; Mitrea, Cika, et al., 2018; Patel et al. 2015; Ray et al. 2020; Sankaranarayanan et al. 2021; J. Wang et al. 2018). By selectively photobleaching a region of interest and tracking fluorescence recovery over time, FRAP provides insights into the diffusion and binding kinetics of molecules inside condensates (Taylor et al. 2019). Faster recovery times indicate high molecular mobility, characteristic of liquid-like condensates, while slow or incomplete recovery suggests gel-like or solid-like material states (Fig. 3B).

Despite its widespread use, FRAP has intrinsic limitations that complicate its interpretation, particularly in the context of condensates. One major challenge arises from the three-dimensional nature of condensates, which makes accurate quantification difficult (Taylor et al. 2019; Wang et al. 2021). Unlike simple solutions where diffusion occurs homogeneously, biomolecular condensates exhibit spatial and temporal heterogeneity, leading to variations in fluidity across different regions and among different molecular components within the same condensate (Boeynaems et al. 2019). Additionally, FRAP is highly sensitive to experimental conditions, instrumentation, and analysis parameters. A fundamental constraint in condensate FRAP studies is that many protein condensates form at small sizes (~ 1 μm), making it inevitable that the bleaching region is comparable in size to the entire droplet. This introduces complications such as finite boundary effects and interfacial resistance, both of which influence the recovery profile and can confound quantitative interpretation (Alshareedah et al. 2021a, b).

The interpretation of FRAP data is also strongly dependent on the diffusion model applied. The diffusion coefficient is typically derived as proportional to the area of the bleached region divided by the characteristic recovery time (Day et al. 2012). However, condensates with viscoelastic properties present additional complexities: the choice of fitting models significantly affects the extracted diffusion coefficients and the quality of fit (Taylor et al. 2019). For example, diffusion coefficients of LAF-1 protein droplets at 80 mM NaCl have been shown to vary by a factor of 5 depending on the chosen model (Taylor et al. 2019).

Given these limitations, FRAP alone is often insufficient to fully resolve the material properties of biomolecular condensates. To overcome its constraints, researchers frequently employ additional biophysical techniques such as fluorescence lifetime imaging microscopy (FLIM) and single-particle tracking (SPT). By integrating these complementary methods, a more complete understanding of condensate dynamics can be achieved, allowing researchers to disentangle the contributions of molecular diffusivity, viscosity, and intermolecular interactions with greater precision.

### Fluorescence lifetime imaging microscopy (FLIM)

Fluorescence lifetime imaging microscopy (FLIM) is an advanced time-resolved technique that quantifies the fluorescence lifetime—the duration a fluorophore remains excited before emitting a photon (Bastiaens and Squire 1999; Fahim et al. 2025) (Fig. 3C). Unlike intensity-based fluorescence methods, which are prone to artefacts from concentration fluctuations and photobleaching, FLIM offers a robust, concentration-independent measure of the local microenvironment within condensates (Fahim et al. 2025). This makes it ideal for probing viscosity, molecular crowding, and environmental changes in these dynamic, droplet-like cellular structures (Becker 2012; Mangiarotti et al. 2023; Ranjit et al. 2018). By exciting samples with a pulsed laser and collecting time-correlated single-photon counting (TCSPC) data, FLIM generates fluorescence decay curves, from which lifetimes are derived via exponential fitting (Becker 2012). Fourier analysis further transforms these data into phasor plots, enabling model-free visualisation and segmentation of lifetime distributions, distinguishing condensate signals from autofluorescence or noise (Malacrida et al. 2021).

FLIM excels in probing the biophysical properties of condensates, bridging in vitro and *in cellulo* studies. For example, it has been used to characterise FUS and TDP-43 condensates, revealing how amyotrophic lateral sclerosis-linked mutations increase viscosity and reduce dynamics, with fluorescence lifetime directly correlating to intra-condensate viscosity (Chung et al. 2022). Similarly, FLIM distinguished liquid-like from solid-like states in Dhh1 condensates (Makasewicz et al. 2024) and tracked structural transitions in α-synuclein amyloid formation, linking lifetime decreases to energy transfer in β-sheet-rich fibrils (Hardenberg et al. 2021). Its sensitivity to molecular crowding and environmental factors—such as pH, ion concentration, and polarity—allows FLIM to monitor viscosity changes and condensate maturation over extended timescales, offering a unified view of condensate evolution across experimental conditions. Another key advantage of FLIM is its independence from fluorophore concentration, which differentiates it from intensity-based measurements that are susceptible to concentration fluctuations and photobleaching (Fahim et al. 2025; Suhling et al. 2015). Unlike FRAP, which relies on diffusion, FLIM directly reports on local microenvironments, enhancing its utility for correlating in vitro and *in cellulo* viscosity data.

However, one major constraint of FLIM is its relatively long acquisition time, requiring stable condensates to avoid motion artefacts, and raster scanning limits its resolution and speed for highly dynamic systems (Ma et al. 2021; Makasewicz et al. 2024). Emerging solutions, such as deep learning–enhanced lifetime prediction from sparse photon data, are reducing these constraints and improving throughput (Kapsiani et al. 2025). While FLIM does not directly measure molecular exchange kinetics like FRAP, its ability to capture biochemical heterogeneity in local microenvironment properties—via lifetime shifts tied to viscosity and compaction changes—makes it a high-resolution, minimally invasive technique for studying condensate material properties across diverse biological contexts.

### Flicker spectroscopy

Flicker spectroscopy quantifies interfacial tension and bending rigidity directly by analysing the spontaneous shape fluctuations of condensates. Unlike fusion-based methods, which infer interfacial tension indirectly through droplet coalescence, flicker spectroscopy extracts interfacial tension and bending rigidity from the fluctuations in the shape of the condensate without requiring perturbation of the system from live-cell or in vitro imaging (Law et al. 2023; Rautu et al. 2017). Originally developed to study lipid vesicles and red blood cells, the technique has long served as a non-invasive tool for measuring mechanical properties in soft matter systems (Fricke et al. 1986; Rautu et al. 2017). Its recent application to study nucleolar surface fluctuations in human cells showed that the technique can probe intracellular material properties using endogenous fluctuations (Caragine et al. 2018). The technique hinges on the thermally driven shape deformations exhibited by condensates, analogous to lipid vesicles and other soft matter interfaces. High-speed imaging (e.g. 25–30 frames per second) captures these fluctuations, enabling an image analysis algorithm to extract condensate outlines and decompose boundary fluctuations into Fourier modes (Fig. 3D). Representing various deformation scales, these modes facilitate the determination of interfacial tension and bending rigidity by fitting corrected fluctuation spectra to theoretical models of membrane elasticity and capillarity (Esposito et al. 2007). In this context, reduced fluctuations correlate with higher interfacial tension, while shape undulations increase as interfacial tension decreases, providing a robust framework for mechanical property quantification. It has been used to assess condensate stiffness in stress granules, revealing distinct mechanical properties between organelle-associated and cytoplasmic condensates (Law et al. 2023).

A significant advantage of flicker spectroscopy lies in its non-invasive, direct measurement of material properties via high-resolution live-cell microscopy imaging (Law et al. 2023). Utilising short imaging bursts, the method minimises disruption, rendering it particularly suitable for live-cell investigations. Its high-throughput nature further enhances its utility, allowing the analysis of thousands of condensates within a single dataset to yield statistically robust characterisations across diverse conditions. Notably, Law and co-workers demonstrated its capacity to distinguish stress granule types based on mechanical property distributions, highlighting its sensitivity to cellular variability (Law et al. 2023). By relying on thermal fluctuations rather than active perturbation, flicker spectroscopy circumvents potential photobleaching-induced cell damage, ensuring that condensate properties are measured in their native state without external influence.

However, flicker spectroscopy requires high-resolution imaging and precise tracking of shape fluctuations, making it challenging to apply in highly crowded intracellular environments. While flicker spectroscopy requires high-resolution imaging, advances in multifocal microscopy and adaptive optics now mitigate crowding artefacts, enabling more accurate condensate shape fluctuation analysis in dense intracellular environments (Laine et al. 2023). Despite these limitations, it remains a promising method for investigating condensate mechanics in physiologically relevant conditions and aligning in vitro measurements with *in cellulo* findings.

Similar to flicker spectroscopy, another emerging method that repurposes standard fluorescence microscopy to study condensate properties is raster image correlation spectroscopy (RICS). RICS extracts diffusion coefficients by analysing fluorescence fluctuations in confocal raster scans, offering spatially resolved maps of molecular mobility without requiring specialised hardware. RICS has been successfully applied to compare molecular diffusion and mobility profiles across nucleoplasmic condensates, revealing distinct material behaviours and demonstrating that diffusion heterogeneity reflects underlying phase organisation (Strom et al. 2017). Recently, RICS was also combined with microelectrophoresis to quantify condensate zeta-potential, viscosity, and diffusion coefficient in vitro (van Haren et al. 2024). These examples showcase the versatility of RICS for probing condensate material properties both in cells and in vitro.

### Brillouin light scattering (BLS) microscopy

Fluorescence-based methods offer a powerful approach to studying the material properties of biomolecular condensates. However, the concentration enrichment in the dense phase can lead to quenching and energy transfer effects that may complicate the result interpretation (Incicco et al. 2023). Therefore, there is an emerging trend of developing different label-free techniques to overcome these challenges and provide more accurate insights into the physical characteristics of these dynamic systems.

Among these, Brillouin light scattering (BLS) microscopy has gained prominence as a label-free, non-contact method for assessing the mechanical properties of biomolecular condensates in vitro and in cells. BLS exploits the inelastic scattering of light by thermally induced acoustic phonons, a population of microscopic acoustic waves, providing insights into the viscoelastic properties of biological samples. By measuring the Brillouin shift and the linewidth related to viscosity, this technique enables the quantification of stiffness and internal fluidity in live cells and tissues (Fig. 3E) (Prevedel et al. 2019). Unlike traditional mechanical probing techniques such as atomic force microscopy (AFM) and micropipette aspiration, which require direct physical contact and can introduce artefacts, BLS allows for non-invasive 3D spatial mapping of condensate mechanics at subcellular resolution.

Recent applications of BLS in condensates have revealed its capacity to distinguish the mechanical differences between liquid-like and more rigid structures. For instance, BLS studies on amyloid-β plaques and stress granules have shown increased Brillouin shifts compared to their surrounding cytoplasm, suggesting increased stiffness within these pathological aggregates (Mattana et al. 2017). Similarly, BLS has been applied to study the mechanical properties of stress granules formed under oxidative stress conditions in mammalian cells expressing ALS-associated FUS variants (Antonacci et al. 2018).

One of the major advantages of BLS is its compatibility with other complementary imaging modalities that enhance the correlation of mechanical properties with biochemical composition. For example, integrating BLS with Raman spectroscopy has enabled researchers to assess both the mechanical rigidity and molecular identity of protein aggregates (Mattana et al. 2017), while integration with optical diffraction tomography (ODT) — a technique for reconstructing 3D refractive index distributions — facilitates quantification of the viscoelastic properties of inhomogeneous compartments within condensates by enabling accurate density correction for Brillouin-derived measurements (Schlüßler et al. 2022).

BLS is largely limited by its diffraction-limited resolution, relatively slow acquisition speed, and the requirement for high irradiances, which may affect sensitive biological samples. Furthermore, commercial availability remains a challenge, as many implementations require custom-built systems (Ibrahim et al. 2024). However, recent developments leveraging artificial intelligence for data processing and super-resolution BLS techniques hold promise for expanding its applicability in cell biology and mechanomedicine.

## Conclusion and outlook

The accurate measurement of biomolecular condensate material properties in live cell environments remains a formidable challenge due to molecular crowding, compositional heterogeneity, and technical artefacts introduced by conventional fluorescence-based methods. While widely used techniques (summarised in Table 1) such as fusion assays and FRAP provide valuable insights into condensate dynamics, they are often constrained by factors including tag-induced perturbations. To date, the progress in applying these methods to condensates in the cellular environment has relied heavily on the use of fusing the protein of interest to fluorescent proteins and optimising the techniques on the FP photophysics. Recent application of the tetracysteine tag/FlAsH-EDT_2_ strategy in varied cellular and condensate systems (Hoffmann et al. 2010; Ng et al. 2025; Ray et al. 2020; Roberti et al. 2007) offers an alternative to traditional FPs by providing a smaller, more specific labelling approach with potentially reduced perturbation to condensate properties. Given the versatility of FlAsH-labelling for both in vitro and live cell labelling and imaging, developing its use with advanced microscopy techniques for probing condensate materials properties is an exciting prospect. Additionally, label-free techniques such as Brillouin light scattering provide a non-invasive means of probing mechanical characteristics, circumventing some of the fundamental drawbacks of fluorescence-based imaging. However, no single method can comprehensively capture condensate behaviour across all relevant physical scales. Future advancements in this field will require an integrated approach that combines high-specificity fluorescence techniques with label-free mechanical characterisation, alongside computational models to bridge in vitro and *in cellulo* discrepancies. By refining and standardising these methodologies, we can move towards a more complete and physiologically relevant understanding of condensate material properties, with broad implications for cell biology and condensate-based biotechnologies.

**Table 1:** Summary of techniques discussed towards the characterisation of biomolecular condensate material properties

Technique	Measured parameter	Fluorescence labelling	Applicable system
Coalescence-based techniques	Viscosity, interfacial tension	Optional	In vitro (Fabrini et al. 2024; Sato and Takinoue 2023) & *In cellulo* (Brangwynne et al. 2009, 2011; Català-Castro et al. 2025)
FRAP	Molecular diffusivity/viscosity, diffusion coefficient	Required	In vitro (Mitrea, Cika, et al., 2018; Patel et al. 2015) and *In cellulo* (Ray et al. 2020; Sankaranarayanan et al. 2021)
FLIM	Molecular packing	Required	In vitro (Chung et al. 2022) and *In cellulo* (Makasewicz et al. 2024)
Flicker spectroscopy	Interfacial tension, bending rigidity	Optional	In vitro (Williamson et al. 2025) and *In cellulo* (Law et al. 2023)
RICS	Diffusion coefficient (spatial/temporal correlation)	Required	In vitro (van Haren et al. 2024) and *In cellulo* (Strom et al. 2017)
Micro-rheology	Viscosity, viscoelasticity (passive/active bead tracking)	Optional	In vitro (Pan et al. 2021; Wei et al. 2017) and *In cellulo* (Català-Castro et al. 2025; Shen et al. 2023)
Differential dynamic microscopy (DDM)	Collective diffusivity, dynamic heterogeneity	Optional	In vitro only (Linsenmeier et al. 2022)

## Data Availability

No datasets were generated or analysed during the current study.
